# Sulfasalazine-Induced Agranulocytosis: A Case Series and Review of Literature

**DOI:** 10.31138/mjr.010124.sia

**Published:** 2024-09-30

**Authors:** Nikita Chettri, Mahabaleshwar Mamadapur, Ramaswamy Subramanian, Dharmarajan Sandhya, Jaidev Kumar B R

**Affiliations:** 1 Department of Pharmacy Practice, JSS College of Pharmacy, JSS Academy of Higher Education and Research, Sri Shivarathreeshwara Nagar, Mysuru, Karnataka, India; 2 Department of Clinical Immunology and Rheumatology, JSS Medical College and Hospital, JSS Academy of Higher Education and Research, Sri Shivarathreeshwara Nagara, Mysuru, Karnataka, India; 3 Department of ENT, JSS Medical College and Hospital, JSS Academy of Higher Education and Research, Sri Shivarathreeshwara Nagara, Mysuru, Karnataka, India

**Keywords:** sulfasalazine, agranulocytosis, rheumatoid arthritis, G-CSF

## Abstract

Sulfasalazine (SSZ) remains a valuable treatment option for Rheumatoid Arthritis (RA), especially in women of childbearing age, and is considered safe in pregnancy and lactation. However, the adverse effects in the form of allergic reactions, rashes, feverishness, and gastrointestinal symptoms are not uncommon and usually resolve on discontinuation of the drug. Despite the potential adverse effects, the occurrences are infrequently reported. Agranulocytosis (ANC < 500 cell/cumm) is a rare complication of SSZ that may be potentially life-threatening. We report two cases of SSZ-induced agranulocytosis after 6 weeks of initiation of treatment for RA despite normal leucocyte counts in the initial phase of treatment. There was complete recovery of the counts following discontinuation, along with the institution of colony-stimulating factors and antibiotics for febrile neutropenia. Notably, Granulocyte Colony-Stimulating Factor (G-CSF) did not produce any adverse effects, and the patients were discharged after their ANC levels returned to normal. It is, therefore, essential to regularly monitor blood counts following the initial treatment.

## INTRODUCTION

Agranulocytosis is defined as an absolute neutrophil count of less than 0.5 × 109/L.^1^ It is an uncommon but potentially life-threatening side effect of sulfasalazine which usually occurs within the initial three months of treatment and resolves within two weeks of drug discontinuation.^2^ SSZ remains a valuable option for mitigating symptoms and slowing the progress of RA. We report two cases of RA with severe agranulocytosis following treatment with SSZ, necessitating G -CSF.

## CASE 1

A 26-year-old female presented with dysphagia, fever with chills, and dysarthria for 3 days. She initially visited an otolaryngologist but had no improvement in her symptoms. Four months prior, she was diagnosed with RA based on symmetrical joint pains and tested positive for Rheumatoid Factor (RF latex >256 IU/ml). As she was lactating then, she was started on SSZ at an incremental dose to a dose of 1500 mg/day, which she tolerated well. In 2017, during her pregnancy, she was also diagnosed with β-thalassemia minor and did not have any significant family history.

On examination, the oral cavity showed a membrane over both tonsils and oral secretions were present. Haemoglobin (Hb) level was 6.9 g/dL, total leukocyte count (TLC) was 220 cells/mm^3^, absolute neutrophil count (ANC) was 10 cells/μL, platelet count was 2.57 L/mm^3^, and erythrocyte sedimentation rate (ESR) was 150 mm/hr. A peripheral blood smear (PBS) showed microcytic hypochromic anaemia along with leukopenia. Serum ferritin was 517.7 ng/ml. Her bone marrow biopsy showed a cellular marrow with a complete absence of granulocytes and granulocyte precursors (agranulocytosis) with increased macrophage activity. The antinuclear antibodies (ANA) test was negative.

**Figure 1.**
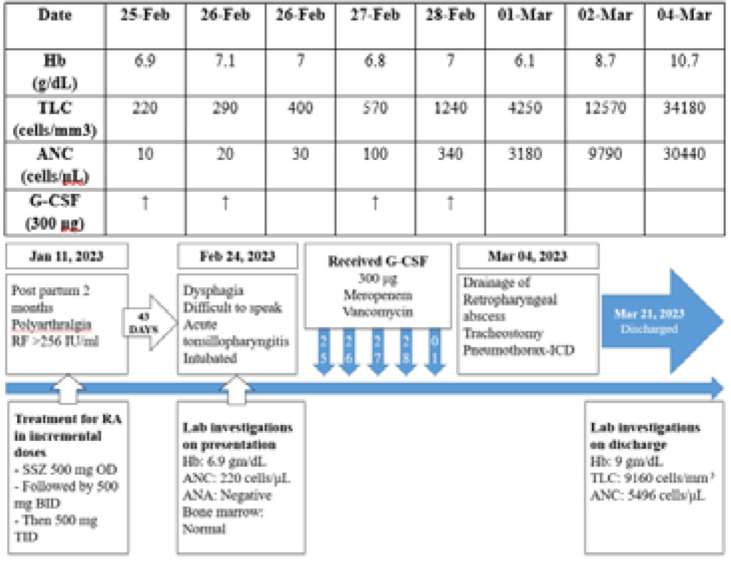
Course of first patient in the hospital.

On day 2, the patient was started on subcutaneous injection of G-CSF 300 μg once daily (OD) for 5 days with meropenem and vancomycin for febrile neutropenia. The patient’s general condition improved on day 6 of treatment with an ANC of 3180 cells/μL. On day 7, the voice hoarseness worsened. CT scan of the neck showed hypodense collection in the right tonsillar fossa and right sublingual space suggestive of abscesses. She later underwent a tracheostomy and incision and drainage of the para pharyngeal abscess. The patient also received two packed red blood cell (PRBC) units. The patient developed dyspnoea during postoperative stay and the X-ray showed right-sided pneumothorax. Her dyspnoea settled with chest tube insertion (ICD). Epiglottis oedema was managed with steroids. The patient was discharged on the 26^th^ day. The patient’s course in the hospital is demonstrated in **Figure 1** below.

## CASE 2

A 29-year-old female patient presented with symptoms of fever, throat pain, swelling, and dysphasia for five days. Two months back, she was diagnosed with seropositive RA. She had hypothyroidism and was on active thyroid replacement therapy. As she was planning a pregnancy, she was started on an incremental dose of SSZ 1500 mg per day.

On examination, the patient had facial puffiness, an ulcer on the right cheek, and induration in the right submandibular area. ENT evaluation showed oral gingivitis and pharyngeal wall congestion, and laryngoscopy was normal. Hb level was 13 g/dL, TLC was 810 cells/mm^3^, ANC was 28 cells/μL, platelet count was 4 L/mm^3^, and an ESR was 150 mm/hr. The PBS indicated normocytic normochromic red blood cells and leukopenia.

**Figure 2.**
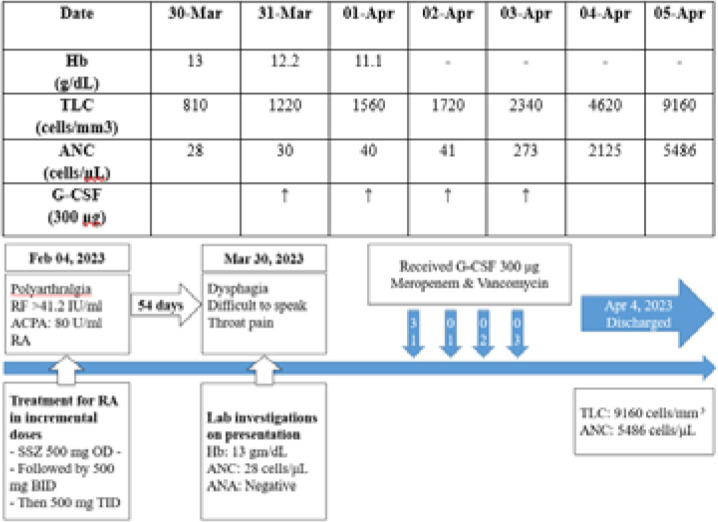
**Figure 1.** Course of second patient in the hospital.

On the second day, the patient was started on subcutaneous G-CSF 300 mcg OD injection and continued for four days along with meropenem and vancomycin for febrile neutropenia. Her overall condition and ANC gradually improved, with the value reaching 5486 (cells/μL) by the seventh day and the patient was discharged. The patient’s course in the hospital is demonstrated in **Figure 2** below.

## DISCUSSION

Sulfasalazine is an effective drug in the treatment of RA. It is often included in the triple-drug regimen in combination with methotrexate and hydroxychloroquine which was very common before the use of biologics.^3^ It can also be used in patients with RA where the use of methotrexate and leflunomide are contraindicated. Sulfa-induced drug reactions are not uncommon and they often manifest with symptoms ranging from nausea, vomiting, loss of appetite, indigestion, headache, and skin rash to severe allergic reactions of which the pathogenesis is not very clear. Usually drug-induced bone marrow suppression is common with most DMARDs. Still, isolated agranulocytosis is rare except with the usage of azathioprine which is due to either thiopurine methyltransferase (TPMT) or the enzyme nudix hydrolase 15 (NUDT 15) mutations.

According to a study conducted in a Swedish population, the incidence of agranulocytosis in patients receiving SSZ for inflammatory bowel disease and RA was estimated to be 1 in 2,400 (0.04%) patients during the first month of treatment, 1 in 700 patients during the second and third months (0.14%), and 1 in 11,200 (0.009%) patients after more than 3 months of treatment.^4^ Whereas, in a similar study conducted by Jick H et al., the estimated risk of agranulocytosis among SSZ users was 6.8/10,000 users. For users of 30 days or less, the incidence was estimated to be 2.9/10,000 users; for users of 31–90 days, it was 3.6/10,000; for users of more than 90 days, it was 1.5/10,000. The risk of agranulocytosis was 7/3781 for subjects treated for arthritis, compared with 0/6286 for those treated for IBD.^5^ The fatality rate of sulphasalazine-induced agranulocytosis was 6.5 %, and the median recovery time for the patient who recovered was 10 days.^6^

**Table 1.** Laboratory parameters of two cases at presentation.

**Laboratory Parameters**	**Case 1**	**Case 2**	**Reference ranges**
Hb (g/dL)	6.9	13	11.5–16.5
TLC (cells/mm3)	220	810	4,000–11,000
ANC (cells/μL)	10	28	2000–8250
Platelet Count (L/mm3)	2.57	4.0	1.5–4.5
ESR (mm/hr)	150	150	0–20
CRP (mg/L)	368.86	221.09	0–5
PBS	Microcytic hypochromic anaemia with leukopenia	Normocytic, normochromic RBC, leukopenia	
Bone marrow biopsy	Cellular marrow with a complete absence of granulocytes and granulocyte precursors (Agranulocytosis) with increased macrophage activity.	Not done	

Hb: haemoglobin; TLC: total leukocyte count; ANC: absolute neutrophil count; ESR: erythrocyte sedimentation rate; CRP: C-reactive protein; PBS: peripheral blood smear.

Furthermore, SSZ can also cause blood dyscrasias, pancreatitis, interstitial nephritis, hepatitis, and hepatic failure. Hence, the American College of Rheumatology (ACR) recommends testing complete blood count at baseline, every 2 to 4 weeks for the first 3 months, every 8 to 12 weeks for 3 to 6 months, and every 12 weeks after that.^7^

SSZ usage in two of our RA cases led to the development of isolated agranulocytosis after > 6 weeks of initiation of treatment, despite monitoring of counts in the initial phase. Both cases had an aggressive course with the development of a para pharyngeal abscess in case 1 and an ulcer on the right cheek in case 2 as a sequela of agranulocytosis. There was however a complete recovery with the institution of G-CSF, broad-spectrum antibiotics, and drainage of the abscess along with tracheostomy and ICD management in Case 1.

SSZ is mainly used to manage inflammatory bowel disease and rheumatoid arthritis. Its mechanism of action is under investigation but is believed to work by blocking the transcription of genes that cause inflammation, such as TNF-alpha, by inhibiting nuclear factor kappa-B. SSZ is a prodrug that is too large to be absorbed in the small intestine and is activated only when broken down by bacteria in the colon. Once activated, it releases 5-aminosalicylic acid, which has anti-inflammatory effects. Additionally, SSZ inhibits osteoclast formation by suppressing the expression of receptor activators of NF-kB ligand (RANKL) and stimulating osteoprotegerin, a natural inhibitor of RANKL.^8^ The postulated mechanisms of SSZ-induced agranulocytosis include immune-mediated sequestration of circulating neutrophils or direct toxicity of the granulocytic precursors in the marrow. Intestinal bacteria metabolise the drug to 5-amino salicylic acid and sulfapyridine. NAT2 and CYP2C9 enzymes catabolise sulfa-pyridine to excretable hydroxylamine whereas, 5 aminosalicylic acid is responsible for the pharmacotherapeutic effects in RA.4 Mutations in NAT2 and CYP2C9 lead to the accumulation of hydroxylamine which causes toxicity. A genome-wide association study (GWAS) conducted on a European population by Wadelius M et al. revealed that those with genetic variants in HLAB*08:01 and HLAA*31:01 had an increased susceptibility to developing sulfasalazine-induced agranulocytosis.^4^ In addition, idiosyncratic skin and lupus-like reactions have been attributed to slow NAT2 Acetylators.

SSZ has been in use for the treatment of RA since the 1930s and its association with agranulocytosis was already reported in 1942.^9^ We reviewed the literature using the PubMed database with the keywords “Sulfasalazine”, “Agranulocytosis”, and “Rheumatoid Arthritis”, up to November 2023. The cases reported in the literature so far are summarised in **Table 2**, case reports with full-text articles were only included.

**Table 2.** Summary of the similar existing reports.

**Study (Year)**	**Duration of Sulfasalazine intake**	**No. of patients**	**Primary Diagnosis**	**Management**	**Outcome**
Cochrane P. et al. (1973)^24^	19 days	1	Ulcerative colitis	Sulfasalazine withdrawn Vigorous treatment with gentamycin (60 mg i.v., 8 hourly), cloxacillin (500 mg i.v., 6 hourly), hydrocortisone (100 mg i.v., 6 hourly), and γ globulin (500 mg i.m. Stat.).	Recovered
Maddocks and Slater (1980)^25^	56 days	1	Active proctitis consistent with Ulcerative colitis	Sulfasalazine withdrawn Intravenous fluids, methylprednisolone, and, based on sensitivity tests, with i.v. fucidin and gentamicin. Also received granulocyte transfusion of 4 x 1010 cells obtained from relatives.	Death
Farr M. et al. (1986)^10^	1 month	1	Ankylosing spondylitis	Sulfasalazine withdrawn	Recovered
		1	Rheumatoid arthritis	Sulfasalazine withdrawn	Recovered
		3	Inflammatory arthritis	Sulfasalazine temporarily withdrawn	Recovered
		3			Recovered
Derry and Schwinghammer (1988)^26^	7 weeks	1	Diverticulitis	Sulfasalazine discontinued by patient	Recovered
				Empiric intravenous antibiotic	
Bliddal H. et al. (1989)^12^	2 weeks	3	Rheumatoid arthritis	Sulfasalazine withdrawn	Recovered
Rui M. et al. (1990)^13^	4 weeks	1	Ulcerative colitis	-	Recovered
Murphy P. et al. (1990)	12 weeks	1	Rheumatoid arthritis	Sulfasalazine withdrawn Amikacin, Pipericillim, Metronidazole, Flucloxacillin, Vancomycin, Ceftazimide, and Amphotericin	Recovered
Palmblad J. et al. (1990)^11^	2 months	1	Rheumatoid arthritis	Amikacin, Cefuroxime rhGM-CSF (250 mcg-2)	Recovered
Gales B. J. et al. (1993)^14^	2 months	1	Rheumatoid arthritis	Sulfasalazine withdrawn Ceftazidine, Aztreonam G-CSF (600 mcg/d)	Recovered
Roddie P. H. et al. (1995)^15^	5 weeks	1	Ulcerative colitis	Gentamicin, GM-CSF (450 mcg)	Recovered
Chan K. et al (2015)^16^	2 months	1	Inflammatory arthritis	G-CSF (0.3 mg/d)	Recovered
Fathallah N. et al (2015)^17^	8 weeks	1	Ulcerative colitis	G-CSF, amikacin, tazocin, corticotherapy	Recovered
Kato M. et al (2016)^18^	-	1	Rheumatoid Arthritis	G-CSF	Recovered
Homsy S. et al (2020)^19^	-	1	Psoriatic arthritis	-	Recovered
Current report	4 months	1	Rheumatoid Arthritis	G-CSF, meropenem, vancomycin	Recovered
	6 weeks	1	Rheumatoid Arthritis	G-CSF, meropenem, vancomycin	Recovered

Farr M et al. studied the side effect profile of SSZ on 200 patients with inflammatory arthritis who were treated for a minimum of one year. Of these patients, 166 had RA. The results indicated that 58% of patients experienced one or more negative reactions to the drug, and 21.5% had to stop taking it. However, 28% continued using the drug but with a reduced dose. The most common side effects reported were gastrointestinal (33%) and central nervous system reactions (19%), but they were not severe. Although some patients experienced serious effects such as neutropenia (2%), thrombocytopenia (1%), and pan-hypogammaglobulinemia (1%), all reactions diminished either when the drug was discontinued or the dose was reduced, in all patients.^10^

The first case of treatment of drug-induced agranulocytosis with recombinant GM-CSF was reported by Palmblad J et al. in 1990.^11^ Various cases have been reported later where drug-induced agranulocytosis was treated with G-CSF. Both of our patients were initiated on SSZ as first-line DMARD with regular monitoring of TLC count. However, both had severe agranulocytosis at presentation and were managed with G-CSF.

## CONCLUSION

The sulfasalazine-induced agranulocytosis in these two female patients, occurring > 6 weeks after initiating the drug, highlights the importance of remaining cognizant of the potential for SSZ to cause agranulocytosis, an infrequent but severe side effect. To prevent unexpected complications, it is essential to monitor blood counts even following the initial treatment regularly. Patients should be educated on the associated symptoms and urged to report any anomalies immediately. Consistent monitoring of blood counts is critical in detecting abnormalities and ensuring patient safety. Therefore, it is recommended that a monitoring plan be implemented that includes regular assessments of blood parameters to guarantee patient safety.
